# Twenty seconds of visual behaviour on social media gives insight into personality

**DOI:** 10.1038/s41598-022-05095-0

**Published:** 2022-01-21

**Authors:** Callum Woods, Zhiyuan Luo, Dawn Watling, Szonya Durant

**Affiliations:** 1 Psychology Department, Royal Holloway University of London, Egham, TW20 0EX UK; 2 Computer Science Department, Royal Holloway University of London, Egham, TW20 0EX UK

**Keywords:** Psychology, Human behaviour, Oculomotor system

## Abstract

Eye tracking allows the researcher to capture individual differences in the expression of visual exploration behaviour, which in certain contexts has been found to reflect aspects of the user’s preferences and personality. In a novel approach, we recorded the eye movements of 180 participants whilst they browsed their Facebook News Feed and employed a machine learning approach to predict each of the self-reported Big Five personality traits from this viewing behaviour. We identify that specific visual behaviours are informative of an individual’s personality trait information, and can be used to psychologically profile social networking site users significantly better than chance after collecting only 20 seconds of viewing behaviour. We discuss potential applications for user engagement during human–computer interactions, and highlight potential privacy concerns.

## Introduction

Tailoring content to appeal to the user’s personality can promote consumer loyalty and engagement^1^. Similarly, appealing to the user’s personality can lead to increased conversion rates during online marketing campaigns, with personality-congruent personalised advertisements leading to up to 50% more purchases compared to non-personalised or personality-incongruent advertisements^2^. As such, the ability to quickly predict the personality of the user is of value to providers who wish to maximise the potential for users to engage with, and relate to, a wide range of services and content.

Online social networking sites (SNS) provide content that is socially and emotionally relevant to the user and enables users to connect, share content and interact with others as part of a personally tailored experience. Machine learning techniques have been successfully applied to records of SNS behaviour to predict aspects of the user’s private traits and attributes, such as their age, gender, political inclination and personality^3^. A recent meta-analysis identified that the self-reported ’Big Five’ personality traits (Openness, Conscientiousness, Extroversion, Agreeableness and Neuroticism)^4^ were the most commonly predicted individual characteristics from online digital traces, and that the Facebook platform was the most common SNS investigated^5^. The meta analysis also found a moderate meta-correlation (0.34) between various digital traces and the Big Five personality scores across 29 independent data sets, illustrating that an individual’s personality is reflected in their online behaviour on Facebook^5^. However, currently existing methods of predicting a user’s personality from SNS engagement require access to the user’s detailed personal content and previous behaviour, often across months or years of use. Due to the volume of data provided by eye tracking, a possible advantage of predicting a user’s personality from their oculomotor behaviour is that accurate predictions may not require past knowledge of SNS behaviour, providing a stand-alone method to evaluate aspects of the user’s personal attributes from a single interaction.

Visual behaviour may provide insight into aspects of an individual’s personality because, as a reflection of the spatial distribution of attention, it is driven in part by our endogenous associations (i.e., is shaped by our previous experiences) with features of the visual scene^6^. We tend to look longer at visual stimuli which we find emotionally salient compared to those that we do not^7,8^, and eye movements are influenced by individual attributes such as aspects of our personality^9^ and our cognitive biases^10^. Furthermore, Bargary and colleagues found, within a sample of 1000 young adults, that an individual’s eye movements during a variety of oculomotor tasks (e.g., following a moving object) provide a distinct and reliable ‘oculomotor signature’ that is stable across time^11^. Subsequent literature builds upon this by identifying that personality traits can be decoded from visual behaviour within both real-world locomotion^12^ and screen-based tasks (viewing a series of static images)^13^. As such, these findings suggest that our visual behaviour provides a signal that reflects a range of our underlying individual traits. However, the results across this literature vary from near perfect prediction of whether someone is low, medium or high for each personality trait in a controlled visual environment^13^ to barely above chance in naturalistic conditions^12^.

Importantly, it is currently unknown whether an individual’s private traits and attributes can be predicted from their visual behaviour upon their own SNS profile. We propose that it may be particularly important to show whether eye movements are informative of personality whilst browsing SNS as it seems increasingly likely that this data will be collected^14^. Thus, whilst users are aware that their eyes are being tracked, they may be unaware of the potential for disclosing personal information simply by how they move their eyes whilst browsing their own social media content. This leads to the key contribution of this paper; to investigate to what extent, if at all, the eye movements made whilst participants’ browse SNS content can be used to predict their personality—a key determinant of differences between individuals^15^. We build upon previous literature by employing a naturalistic stimulus (each person viewing their own Facebook News Feed), and testing a large sample of individuals. It is not a foregone conclusion that eye movements upon SNS content will be informative of the individual’s personality as we are varying both the low-level salience of the content (e.g., spatial frequencies, contrast), as well as the semantic content. These changes in the stimulus across participants may induce random variance that reduces or entirely masks the signal provided by eye movements.

In summary, we allowed participants to browse their own Facebook News Feed^16^ section whilst tracking their eye movements, and employ a machine learning approach to predict whether they score low, medium or high on each of the Big Five^4^ personality traits. We formed five sets of predictor variables (in machine learning terms, feature groups) that each describe a particular aspect of the participant’s oculomotor behaviour (outlined in section “Feature engineering”). We then independently assessed the relative insight provided by each predictor variable set into each of the Big Five personality traits. We chose to explore visual behaviour upon the Facebook platform firstly because its prevalence makes it a relevant real-world stimulus and secondly because, as described above, behaviour upon Facebook is linked to a wide range of personal attributes^5^. Finally, we chose to predict the Big Five personality traits because of their relevance to targeting users with personalised marketing campaigns^2^, and their known association with behaviour upon Facebook^5^. In contrast to previous literature, our aim is to investigate what information might be contained in the eye movements alone, without taking into account details of the content of the personal SNS feed.

## Methods

### Participants

Two hundred and nine participants were recruited from an undergraduate university cohort ($M_{age} = 20.45$, $SD_{age} = 3.38$, 169 Female) with age ranging from 18 to 51 years. All participants had normal, or corrected to normal vision and owned a Facebook account. Demographic descriptors were not used as variables in this study. Participants took part in exchange for course credits or a $\pounds$5 reward. Twenty-nine participants (25 female) were excluded due to software failure, or having less than 80% valid eye position samples. Of the remaining 180 participants, 161 reported using the Facebook platform daily or weekly with 14 reporting monthly usage and five yearly. All participants provided written informed consent prior to the experiment. All experimental protocols were approved by the Royal Holloway, University of London Research Ethics Committee. All methods were performed in accordance with the ethical guidelines and regulations of the Declaration of Helsinki.

### Eye tracking

Each participant viewed their personal Facebook News Feed using the Internet Explorer browser (full-screen) upon a 23-inch TFT monitor (1920 × 1080). The viewing distance was 60 cm, and eye movements were collected using a Tobii TX300 infrared eye tracker sampling at 300 Hz, allowing for free movement of the head. Using Tobii Studio software, a five-point calibration procedure was followed, with the experimenter conducting a visual inspection of calibration accuracy before the task started. Stimuli were presented and data was collected within Tobii Studio. Eye movements can be segmented into periods where the eye is kept stable upon the visual scene (fixation) and information is available for cognitive processing^17^, and into periods where the eye is moving rapidly to a new location and less can be processed from the visual scene (saccades). The Tobii Studio default I-VT filter (Window length: 20ms, Velocity threshold: 30 °C, Minimum fixation duration: 60ms) was used to detect fixations and saccades.

There are many different ways to characterise visual behaviour and previous attempts within the literature broadly fall under one of two categories. One method is to describe the statistical properties of these eye movements themselves (e.g., the mean duration of the fixation or saccades, as in^9,12^). Such statistics capture information about how the individual moves their eyes across the visual scene as a whole (e.g., the participant may make frequent, short fixations interspersed with large saccades). In contrast, the other frequently used method is to describe the distribution of fixations across multiple regions with different types of content, which has been used successfully by researchers^7,8^. This is achieved by segmenting the visual scene into different areas of interest (AOI) and describing the participant’s fixation behaviour within each region. This captures information about how the participant attends to different regions or types of content within the visual scene (e.g., do they look longer at videos rather than text). It is currently unknown whether the manner of describing visual behaviour (i.e., statistical descriptions or AOI-based metrics) influences the accuracy of such classifications. In the feature engineering section “Feature engineering” we describe how we create both statistical and AOI-based descriptions of visual behaviour.

### Visual stimuli

Each participant viewed (scrolled through) their own Facebook News Feed page for one minute, resulting in a unique visual scene for each individual. We asked participants to view content as they would usually, with two caveats: to avoid messaging other users, and avoid navigating away from the News Feed section of their Facebook account. Participants were allowed to engage with interactive elements (e.g., click ’like‘ or comment) provided this would not navigate them away from the News Feed, and could use the mouse and/or the keyboard to scroll. The News Feed section hosts a constantly updating list of content displayed within a dynamically generated, scrolling central column. Example items include a friend’s status updates and shared content, along with advertisements and posts from public groups (for full details, see^16^). The Facebook platform provides several constraints upon the location of visual stimuli that are common across participants. In particular, the dynamically generated content is always displayed within bounding boxes of varying size (depending upon the content; e.g. multiple images, video, or text). Each bounding box is annotated with other user’s reactions (likes, comments, etc.) directly below the content, and provides the user with space to engage in reacting to the content themselves (not used within this experiment). As such, this elicits a typical viewing pattern of observing a piece of content, followed by viewing other users’ reactions to that content.

### Labelling strategy

**Figure 1:**
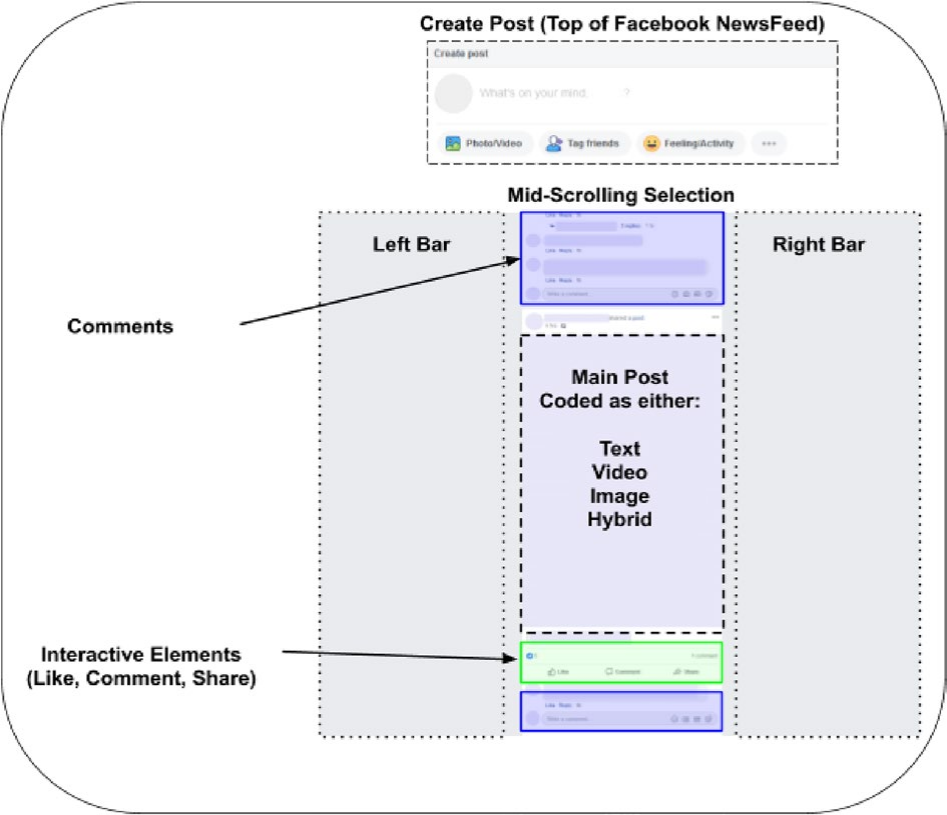
Labelling strategy for Facebook News Feed content. The content categories are ‘Create Post’, ‘Text Content’, ‘Image Content’, ‘Video Content’, ‘Hybrid Content’, ‘Interaction Elements’, ‘Comments’. Each denotes a unique type of content, and allows us to capture visual behaviour in response to this category of visual stimuli. Hybrid Content refers to text overlaid upon an image. Coloured overlays are illustrative and were not displayed to participants.

Using in-built Tobii Studio functionality we obtain a record of the full web page content viewed, on to which visual behaviour has been mapped. Each web page is the product of concatenating multiple screens’ worth of content that has been explored by the individual whilst scrolling through their own News Feed. We found that the software did not always capture the full one minute viewing duration, but reliably captured over twenty seconds. To ensure all participants were directly comparable, each participant’s content was cropped to represent only the first twenty seconds of viewing behaviour. To protect the viewers’ and their friends’ anonymity we obscured all content by blurring and, from earlier piloting, identify seven key types of content (‘Create Post’, ‘Text Content’, ‘Image Content’, ‘Video Content’, ‘Hybrid Content’, ‘Interaction Elements’, ‘Comments’) that frequently occur across participants (Fig. 1). Here, ‘hybrid’ refers to content whereby an image is overlaid with text. These aspects are manually labelled using the free software LabelImg^18^.

### Questionnaire materials

We collected demographic information from participants, including their age, sex and personality traits using the NEO-FFI 60-item inventory^4^. For each personality trait, a score between zero (minimum association with the trait) and 48 (maximum association with the trait) is calculated.

### Machine learning

Personality is often thought of as categorical, for example we say that someone is ‘low’ or ‘high’ upon Extroversion. Furthermore, studies that use mediation analysis to understand the link between personality and other variables will often group individual’s as belonging to the ‘low’, ‘medium’ or ‘high’ category of each of the personality traits^19^. Motivated by this, and following conventions established within previous machine learning literature^3,12,13^, we approached the prediction of personality traits as a supervised classification problem by splitting each trait into three categories (low, medium, high) using an quantile-based binning strategy. Our binning did not result in exactly equal allocations due to discrete scores, thus, as a baseline, we report the highest accuracy and $F1_{macro}$ score possible by predicting all examples to be the majority class. The $F1_{macro}$ score can range from 1 (perfect score) to 0 and was chosen as it equally weights the classifier’s recall ($\frac{True \, Positives}{True \, Positive + False \, Negatives}$) and precision ($\frac{True \, Positives}{True\, Positives + False \, Positives}$) across the three categories (‘low’, ‘medium’, ‘high’). This prevents a classifier from scoring highly by only learning that one category occurs more frequently than the others, which is an advantage over reporting accuracy alone.

**Figure 2:**
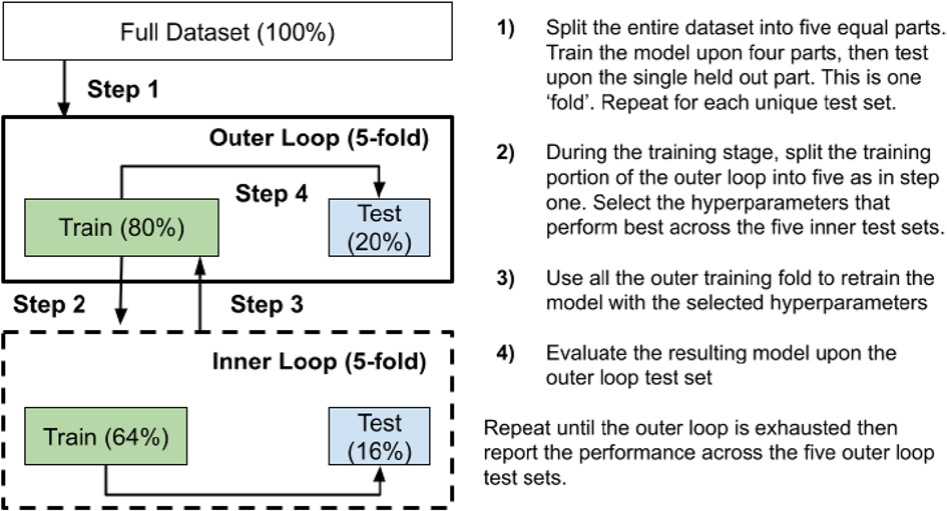
Evaluation strategy for machine learning models. Using a nested cross-validation strategy ensures that the model is always tested upon ’out of sample’ data it has never seen before. In the outer loop we assess the model’s performance. In the inner loop we try to find hyperparameters (settings for the model) that perform well within the training data.

We apply k-nearest neighbours^20^, ridge classification^21^ Support Vector Machines^22^, and naive Bayes classifiers^23^. These algorithms were chosen because of their ability to learn from relatively few samples (i.e., compared to a neural network), and to represent different ways (e.g., linear, non-linear and probabilistic) of learning from the provided data. We standardized each feature independently and within the cross-validation scheme to avoid information about the out-of-sample data leaking into data used to train the model^24^. This ensures each feature has a mean of zero and standard deviation of one. We utilise a nested (outer: five-fold, inner: five-fold) cross-validation procedure, selecting hyper parameters upon the $F1_{macro}$ score metric within the inner loops and collecting accuracy and $F1_{macro}$ scores across the outer loops. This validation scheme is illustrated in Fig. 2. We chose to use a five-fold scheme in both the inner and outer loops as this adheres to best practices for measuring model performance^25^ whilst ensuring that the test sets of the outer fold (36 instances) and inner fold ($\sim$29 instances) are well populated. This is important as having too small of a test set reduces the granularity of the performance estimate (i.e., by constraining the number of values the evaluation procedure can produce).

For models that have accuracy scores greater than baseline and an $F1_{macro}$ score above .33 (chance), we calculate the probability of finding the $F1_{macro}$ result by chance using label permutation^26^, and correct these p-values for multiple comparisons using the Benjamini Hochberg (BH) procedure^27^. Corrected alpha values below .05 are considered significant. Analysis is conducted using the Scikit-Learn library^28^.

### Feature engineering

In a novel approach, this work will be the first to assess the relative capability of using visual metrics that reflect spatial attention across broad categories of stimuli to accurately predict levels of the Big Five personality traits whilst participants view their own SNS content. Specifically, the algorithms are assessing the relative performance of visual metrics that reflect spatial attention, against more general statistical descriptions of eye movements. To understand which of these measures best predict each personality trait, we create separate groups of metrics (‘feature groups’—see Table 1) and evaluate the performance of the models trained upon them. Each feature group is based upon the first twenty seconds of viewing behaviour. Our first four feature groups explore area-of-interest (AOI) based metrics, whereby visual behaviour is grouped in correspondence to the category of content viewed upon the web page. Our fifth group is composed of statistical descriptions of eye movements that are not linked to any particular content. As a final sixth group, we introduce a control feature group describing the proportion of the page occupied, and frequency of occurrence, for each type of content labelled on the Facebook page. This allows us to understand if our oculomotor data is offering insight into each personality trait above and beyond that available from the characteristics of the user’s Facebook News Feed (according to the labelling strategy, as outlined in section “Labelling strategy”). We did not explore whether including age or sex as inputs to the machine learning model improves performance due to our sample’s narrow age range and primarily female cohort.

**Table 1:** Feature groups (evaluated independently).

Feature group	Number of features
AOI	21
AOI proportional	21
AOI with frequency	28
AOI proportional with frequency	28
Eye movement statistics (EMS)	15
Page content info	14

*AOI* Area of Interest Based.

#### AOI-based visual metrics

For each AOI category, we calculate the total fixation duration (TFD), number of fixations, and time to first fixation (TTFF, defined as the time to fixate the first example of the AOI category) values. There are seven content categories (‘Create Post’, ‘Image’, ‘Text’, ‘Video’, ‘Hybrid’, ‘Comments’, ‘Interaction Elements’). For each participant the visual scene is different, leading to a varying proportion of the page being accounted for by each content category across participants. Furthermore, not all categories may be present for a particular participant. To ensure the predictor variables are in a machine friendly format (i.e., do not have missing values) when a content category never occurs for a particular participant (e.g., no videos appeared on their News Feed section within the first twenty seconds), they receive zero for TFD and number of fixations, and the maximum duration of 20 s for TTFF.

However, more can be done to capture additional information about the participant’s viewing behaviour. For instance, by including the frequency of occurrence for each AOI type we can provide information about the extent to which a participant had the opportunity to encounter a type of content (e.g., did they receive zero fixation duration/number because they did not view a content that was present, or because it was never there?). Additionally, as stimuli size varies, some visual metrics (TFD, number of fixations) can be influenced by the amount (proportion) of the page occupied by each category. For example, if the page is covered in text, it is perhaps less surprising that the individual spent a longer duration than most viewing text. To address this, we can re-weight the TFD and number of fixation metrics by the proportion of the page each category (e.g., image, video) occupies.

As such, we investigate whether incorporating this additional information influences classifier accuracy by creating a two sets of predictor variables that have been either supplemented with the frequency of occurrence for each AOI type (denoted by ‘with Frequency’ in Table 1), or where the TFD and number of fixations on each AOI have been corrected for the proportion of the page the AOI accounts for (denoted by ‘Proportional’ in Table 1). Independently evaluating each of these techniques for describing visual behaviour allows us to gain insight into whether accounting for the aforementioned factors influences the ability of the classifier to make accurate predictions.

#### Non-AOI based visual metrics

Inspired by previous literature^12,29^, we create a separate set of metrics that represent overall statistical descriptions of fixation and saccadic behaviour across the Facebook News Feed. We name this group of features ‘Eye Movement Statistics’. We consider fixations to have two attributes (frequency, duration) and saccades to have three attributes (frequency, duration, amplitude). For the frequency attribute we calculate the count (number of instances). For non-frequency attributes we calculate the sum, mean, standard deviation and interquartile range. We also include the mean progress in vertical screen-based coordinates per second (pixels per second) across the viewing duration, as an index of how quickly the participant progresses through their exploration of the web page. This creates a total of 15 features in the statistical feature group (summarised in Table 2).

**Table 2:** Statistical eye movement features.

Event	Property	Metrics
Saccade	Frequency	Count
	Duration	Sum, mean, standard deviation and interquartile range
	Amplitude	Sum, mean, standard deviation and interquartile range
Fixation	Frequency	Count
	Duration	Sum, mean, standard deviation and interquartile range
Vertical progress	Pixels	Pixels per second

Finally, to understand the insight provided by knowing the page content alone (and not the visual behaviour), we included a control feature group consisting of the proportion of the page occupied, and the frequency of occurrence, for each content category (14 features).

## Results

### Personality questionnaire distribution

Our personality questionnaire data contained one participant with one missing questionnaire item response, which was imputed as the mean of the remaining trait-congruent questionnaire items. Each trait has 12 relevant questionnaire items, and within our sample the internal consistency ranged between ’Good’ ($a=0.710$, Openness) and ’Very Good’ ($a=0.869$, Conscientiousness). This illustrates that the questionnaire scores are reliable within our sample. The Shapiro-Wilk test for normality identifies that trait Conscientiousness scores show evidence of being non-normally distributed (W = 0.974, p = 0.002), and as such these scores may not be representative of the general population. No further traits demonstrated evidence of being non-normally distributed. Descriptive statistics for all traits, after splitting into low, medium and high categories, are presented in Table 3.

**Table 3:** Descriptive statistics for big five personality trait scores (out of 48) split by category.

Label	Low	Medium	High	Support
	Mean (SD)	Mean (SD)	Mean (SD)	[Low, medium, high]
Openness	23.95 (2.87)	30.37 (1.48)	36.74 (3.44)	[59, 49, 72]
Conscientiousness	20.28 (4.73)	29.94 (2.18)	37.38 (2.83)	[53, 62, 65]
Extroversion	21.97 (3.18)	29.25 (1.51)	35.05 (2.92)	[59, 48, 73]
Agreeableness	24.41 (3.45)	30.87 (1.25)	37.37 (2.90)	[58, 52, 70]
Neuroticism	17.12 (3.98)	26.37 (1.69)	34.63 (3.28)	[58, 59, 63]

### Social media content and visual behaviour

In our cohort the most frequent type of content to appear on a user’s Facebook News Feed were interaction elements (‘like’, ‘share’, etc). Since these accompany each post, they also let us know that each participant viewed roughly 2–4 posts within the twenty second viewing duration. We report the average total fixation duration and the number of fixations on each AOI type (averaged over participants where the content was shown) in Table 4.

**Table 4:** Frequency and fixation behaviour for each area of interest (AOI) category.

Category	AOI frequency	Fixation duration (ms)	Number of fixations
	Mean (SD)	Mean (SD)	Mean (SD)
Comments	0.88 (0.95)	844 (822)	3.55 (3.89)
Hybrid*	0.59 (0.87)	2210 (1586)	10.01 (7.17)
Image	1.83 (1.33)	2316 (1517)	10.12 (6.58)
Text	2.08 (1.70)	2907 (1919)	13.32 (8.81)
Video	0.69 (0.87)	1584 (1496)	6.65 (6.19)
Create post	1 (0)	258 (392)	1.07 (1.31)
Interaction	3.13 (1.59)	642 (610)	2.74 (2.29)

*An image overlaid with text, typically in a ’meme’ type format.

### Classification results

The best performance achieved across the feature groups for each personality trait is summarised in Table 5. All significance values reported in this section are adjusted for multiple comparisons using the Benjamini Hochberg procedure^27^.

**Table 5:** Best classifier performance statistics for each personality trait.

Trait	Feature group	$F1_{Macro}$ (SD)	Accuracy (SD)	Baseline
	(Algorithm)			$F1_{Macro}$ (Accuracy)
Openness	EMS (SVM)	0.346 (0.016)*	41.7% (3.0)	0.19 (40.0%)
Conscientiousness	AOI proportional (ridge)	0.398 (0.079)*	42.8% (8.5)	0.18 (36.1%)
Extroversion	EMS (ridge)	0.476 (0.051)***	49.4% (5.4)	0.19 (40.6%)
Agreeableness	Page content info (ridge)	0.340 (0.058)	38.9% (6.3)	0.19 (38.9%)
Neuroticism	AOI (Naive Bayes)	0.334 (0.072)	35.6% (6.7)	0.17 (35.0%)

*p < 0.05, **p < 0.01, ***p < 0.001.

*AOI* Area of Interest, *EMS* Eye Movement Statistics, *Ridge* One-vs-Rest Ridge Classification, *KNN* K-nearest neighbors, *SVM* Linear support vector machine.

#### Eye movement statistics

For the Eye Movement Statistics feature set we identify that the personality traits of Openness and Extroversion can be predicted significantly better than chance, and for Extroversion this is achieved across multiple algorithms. The best performance for Openness ($F1_{macro}=0.346, Accuracy=41.7\%$) comes from a linear support vector machine classifier. The best performance for Extroversion ($F1_{macro}=0.476, Accuracy=49.4\%$) comes from using a ridge classifier. We note that the accuracy performance for trait Openness is only marginally better than what can be achieved by classifying all instances as the most frequently occurring category (baseline accuracy $Accuracy=40.6\%$).

#### AOI feature sets

Using the area of interest based descriptions of visual behaviour, the personality trait of Conscientiousness was predicted significantly better than chance using either the AOI ($F1_{macro}= 0.400, Accuracy=42.2\%$), or AOI proportional feature set ($F1_{macro}= 0.398, Accuracy=42.8\%$). We note that this represents a modest improvement over what can be achieved by classifying all instances as the most frequently occurring category (baseline accuracy $Accuracy=36.1\%$), or through knowing only the page content information alone ($F1_{macro}=0.391, Accuracy=40.0\%$).

#### Page content information

For the control feature group of page content information, we find that no traits were able to be predicted significantly above chance.

#### Summary

Across the feature groups the traits of Openness, Conscientiousness and Extroversion were able to be predicted significantly better than chance. No feature group gave significant insight into the personality traits of Agreeableness or Neuroticism. We further explore the significant results presented within this section by investigating the classifier’s performance within each category ([low, medium, high]) in the next section.

### Exploratory analysis: classifier performance by trait category

For each significant model (i.e., with an alpha $<0.05$ after BH correction), we evaluate the *F*1 score for each trait category (low, medium, high) as shown in Table 6. To aid the reader, the standard deviation represents how stable the model’s performance was across the five outer folds (i.e., how much performance varied with different training/test sets).

It is immediately clear that the classifier for trait Openness is performing very poorly for individuals who are average scorers (i.e., those in the medium category). Alongside the earlier remark upon only being marginally more accurate than our baseline, this leads to us deciding that the result for trait Openness should not be interpreted further.

For Extroversion, the ridge classifier performs progressively better as we move from the low, to medium, and finally to the high category. It has a similar *F*1 scores across each trait category, demonstrating a balanced classifier. In contrast, the support vector machine based classifier shows a dip in performance when predicting the medium group, demonstrating an imbalance across the categories.

For Conscientiousness, the Ridge classifier based upon the AOI proportional features has similar *F*1 scores for both the medium and high categories, and substantially worse performance for the low category. The classifier’s performance is most stable when predicting the high category, and most variable when predicting the medium category. For the Ridge classifier built upon the AOI feature set, the performance progressively improves with the quantile category ($low<medium<high$) and is also most variable for the medium category. That each classifier performs markedly worse when predicting the low category for Conscientiousness is intriguing. To understand this further, we calculated how similar participants are within each category when responding to the twelve questionnaire items used to calculate the trait score. For Conscientiousness, the average euclidean pairwise distance between participants becomes smaller (participants respond more similarly) as the quantile-based category increases from low (4.04) to medium (3.9) and high (3.5). As such, we propose that individual’s scoring low upon trait Conscientiousness represent a more diverse (less homogeneous) cohort than high scorers, which may result in a more challenging classification task.

**Table 6:** Classifier performance by personality category for significant models.

Trait	Feature group	F1 score (SD)			Accuracy (%)
	(Algorithm)	Low	Medium	High	
Openness	EMS (SVM)*	0.421 (0.067)	0.090 (0.075)	0.527 (0.045)	41.7
Extroversion	EMS (Ridge)***	0.418 (0.083)	0.481 (0.135)	0.529 (0.092)	49.4
	EMS (SVM)*	0.438 (0.070)	0.338 (0.093)	0.471 (0.067)	43.9
Conscientiousness	AOI Proportional (Ridge)*	0.219 (0.054)	0.493 (0.158)	0.482 (0.076)	43
	AOI (Ridge)*	0.284 (0.108)	0.370 (0.188)	0.546 (0.099)	42
	Page Content Info (SVM)^†^	0.34 (0.101)	0.40 (0.091)	0.43 (0.059)	40

*p < 0.05, **p < 0.01, ***p < 0.001 corrected via Benjamini–Hochberg procedure. ^†^Included for comparison,

*Ridge* One-vs-Rest Ridge Classification, *KNN* K-nearest neighbors, *SVM* Linear support vector machine, *AOI* Area of Interest, *EMS* Eye Movement Statistics.

## Discussion

Online social networking sites (SNS) provide a rich and ecologically valid visual experience with a variety of content and information being presented. Previous literature has illustrated that various aspects of a user’s online behaviour upon SNS, such as the distribution of ‘likes’ upon Facebook^3^, or content of text-based posts upon Twitter^30^, can be used to predict aspects of an individual’s personality. In a novel approach, we present evidence that an individual’s pattern of eye movements, whilst browsing their own Facebook News Feed section, is informative of aspects of their personality (Extroversion and Conscientiousness).

### Main findings

Our primary finding is that the eye tracking based techniques discussed within this paper provide a novel and non-intrusive method of predicting an individual’s Extroversion and Conscientiousness category (low/medium/high) from a single twenty second interaction. This is a substantially shorter time scale than employed within previous literature (e.g., 20 s versus Hoppe et al.’s  12.5 mins^12^ and Berkovsky et al.’s 9.2 mins^13^). It is also, to the authors knowledge, the first time that personality has been predicted from eye movements within this type of stimulus. This finding may support the development of socially aware human–computer interfaces as users’ personalities and visual behaviours are both associated with distinct information-seeking characteristics^31^. Finally, we have discovered that trait Extroversion can be predicted to a greater extent than trait Conscientiousness. Notably, the classifiers predicting trait Conscientiousness vary in their performance across the low, medium and high categories—with the lowest performance when predicting low scorers, and the most success in predicting high scorers. A possible explanation for the varying performance across the categories also comes from the nature of the trait itself, with Conscientiousness being positively associated with task completion^32^, and adherence^33^. Thus, we would expect Conscientiousness to influence both an individual’s visual behaviour, and their completion of the sixty-item personality questionnaire; with high scorers being more methodical (thus exhibiting similar behaviour) and low scorers being less principled (thus exhibiting more varied behaviour) in their approach. To explore this, we calculated how similar participants are within each Conscientiousness category when responding to the twelve questionnaire items used to calculate trait Conscientiousness. Our results support this interpretation, with the average euclidean pairwise distance between participants becoming smaller (participants respond more similarly) as the quantile-based category increases from low to high. As such, we propose that individual’s scoring low upon trait Conscientiousness represent a more diverse (less homogeneous) cohort than high scorers, which is reflected within the labelling strategy and may result in a more challenging classification task.

Interestingly, whilst we found we were able to predict trait Extroversion and Conscientiousness, we were unable to classify participants substantially better than chance for trait Openness, Agreeableness, or Neuroticism within our paradigm. Therefore there appears to be a performance trade-off with measuring visual behaviour over diverse stimuli upon such short time scales when comparing to results from previous literature^12,13^. A direction for future research is to evaluate how predictive accuracy varies with both longer, and shorter, recording durations. We suggest that our findings are most similar to^31^ who investigated whether specific personality traits influence how individuals seek out and process information in information seeking tasks (i.e., whilst using an online search engine). The authors investigated factual, interpretative and exploratory information seeking paradigms and found in all three that Extroversion, Agreeableness and Conscientiousness correlated with the number and total duration of fixations expressed by the individual. In contrast, Openness and Neuroticism were not correlated with any of the measured eye movements. Therefore, if we conceptualise browsing Facebook as a information search task, it is perhaps not surprising that our results indicate that Extroversion and Conscientiousness were able to be predicted significantly better than chance, whilst Openness and Neuroticism were not. This leaves the contradictory finding for Agreeableness, which was not predicted significantly better than chance within our study, yet was found to significantly correlate with eye movements in information search tasks^31^. Agreeableness is likely to influence the individual’s behaviour when choosing whether to accept a particular source of information during a search task, which effectively biases the decision of when to accept that the search goal has been fulfilled and the task has been completed. However, whilst browsing Facebook in this study the participants were engaged in a free-viewing task and not searching for a set goal (i.e., piece of information) and there was no explicit objective to meet. As this is not a directed search, there was no need for participants to choose when to stop and accept the information as sufficient to fulfil the objective, which may be why the trait of Agreeableness was found within previous literature^31^, but was not replicated within this study. Overall, our study’s results suggest that browsing the Facebook News Feed is similar to information search tasks in reflecting trait Extroversion and Conscientiousness, but our design lacked the acceptance criterion that we speculate may be needed for the eye movements to be influenced by the individual’s Agreeableness. This provides a key direction for future research, as experimentally manipulating the browsing task would allow the researcher to empirically investigate if the inclusion of an acceptance criterion is essential for trait Agreeableness to be accurately predicted from visual behaviour.

### Types of SNS content that are predictive of personality

As the perceived task and type of content influences the expression of visual behaviour^34^, we sought to understand how to best characterise visual behaviour in a way that reflects (is predictive of) personality. Within our paradigm, statistical descriptions of visual behaviour that are not tied to any particular content are more informative of trait Extroversion than descriptions of visual behaviour derived from responses to a particular category of stimuli (e.g., AOI-based metrics). Together, this illustrates that statistical descriptions of oculomotor events are informative of trait Extroversion within extremely short time periods, even when each participant views a diverse range of visual stimuli. Our finding of Extroversion being linked to visual behaviour upon SNS content also expands upon the previous work of Rauthmann et al.^9^, who found that in abstract video-based stimuli Extroversion was linked to visual behaviour (being predicted by shorter dwelling times). Our finding could relate to the strong links between Extroversion and sociability^35^, which, given the nature of our social media stimuli, may have provided relevant visual content for evoking trait-congruent visual behaviour. That Extroversion appears to be rapidly manifested in statistical descriptions of oculomotor behaviour whilst viewing SNS content has substantial consequences for the application of personality-detection within applied settings, as this implies it is not essential to know the exact stimuli being presented.

In a novel contribution to the literature, we identify that AOI-based metrics outperform statistical descriptions of visual behaviour when predicting trait Conscientiousness. Our results suggest that, when viewing SNS content, trait Conscientiousness is reflected in the way that the individual distributes their attention across different types of content within the visual scene. In considering why conscientiousness is the only trait presenting better results for the new AOI features (in comparison with the EMS results) we note that Conscientiousness is related to the ability for self-regulation during effortful control^36^, with individual’s scoring higher upon Conscientiousness being more likely to maintain a consistent approach to the given task. In our paradigm, the task was to view their own Facebook News Feed, which provides the ongoing ability for the participant to decide to receive a new stimuli during the session (e.g., ‘Do I look at the comments, or keep on scrolling?’). Thus, it may be that the participant’s level of Conscientiousness influenced their style (e.g., systematic or more chaotic) of visually exploring the content, leading to Conscientiousness being reflected within the distribution of the participant’s visual behaviour across the different content categories.

However, our features were not informative when attempting to predict the remaining personality traits (i.e., the EMS and AOI-based descriptions of visual behaviour held little information above and beyond knowing the content upon the page, which itself was not informative). This appears to conflict with some previous findings such as Berkovsky et al.^13^, who showed that characterising visual behaviour in response to multiple static images can be highly informative of trait personality ($>61\%$ accuracy upon the Big Five personality traits). The difference in results may be attributable to methodological differences. In Berkovsky et al.^13^, visual behaviour was described according to each image seen, which due to the images being identical across participants, were directly comparable. This allows the reasonable assumption that observed variances in visual behaviour between participants are driven by the individual differences, rather than the visual properties of the image^37^. In contrast, our AOI categories represent not a single identical image, but a diverse range of content, and items within a single category may vary in colour, spatial frequencies, subject matter, and more. Whilst this accurately reflects the complex variety of visual and social contexts present upon a fully-featured SNS platform, the expression of visual behaviour is influenced by the properties of the visual stimulus^6,34,37^. As such, our design is likely to have introduced a substantial amount of variance in visual behaviour not directly linked to the user’s personality, which increases the difficulty of the classification problem and may have led to reduced performance. This raises questions regarding whether our results are directly comparable to studies utilising static free-viewing designs, and further suggests that models built upon descriptions of oculomotor behaviour in response to the static free viewing of images may not generalise well within applied SNS settings.

Finally, our choices for the AOI categories were informed by tasks identified as driving distinct visual behaviour (e.g., reading text, searching an image or watching a dynamic scene^34,38^), and aimed to capture visual behaviour in relationship to sufficiently broad categories as to be reasonably comparable across the majority of participants, whilst remaining sufficiently distinct to reflect a unique category of visual behaviour. As we kept our descriptions of visual behaviour broad (regarding labelling of AOIs), the outlined technique could be applied to any web page and this is a direction for future research. However, we note that alternative category choices may lead to improved (or reduced) performance in classifying personality from visual behaviour. Future research may wish to explore which content categorisation schemes best capture trait-congruent visual behaviour.

### Practical implications

Past research has suggested that tailoring a product’s advertising to appeal to an individual’s personality can lead to increased conversion rates during online marketing campaigns^2^, and promote consumer loyalty and engagement^1^. As such, it is desirable to be able to understand the personality of the user in order to maximise the potential for presenting them with engaging human computer interactions. However, current methodologies for evaluating personality either require extensive previous knowledge about the user’s past interactions^1,5^, or are disruptive to a user’s enjoyment of the experience (e.g., a user may not wish to conduct a questionnaire before engaging in an interaction). Whilst the technology described here may not yet be ready for practical applications, the ubiquity of eye tracking devices is growing^14^. This is especially relevant given that our research suggests it is not essential to know the exact stimuli being presented to the individual (e.g., as with our findings for Extroversion) when predicting their personality from visual behaviour. This reduces the demand for rigorous labelling and processing of the users’ social media content, and may provide a privacy-preserving method of implicitly assessing an individual’s personality.

### Summary

To conclude, this study explored the ability for visual behaviour upon an SNS site to give insight into an individual’s personality, in a situation where the classifier has no previous knowledge regarding the user’s past behaviour upon the SNS platform. We demonstrate that within a single twenty second encounter aspects of the users personality can be predicted significantly better than chance. This highlights the possibility of a future where, with additional development, a provider may be able to tailor the presentation of its services or products to the user’s attributes within a very short time frame. However, as the current performance of these classifiers is modest, there may be situations in which visual behaviour metrics can be combined with existing data sources to increase performance when predicting personality traits. For example, previous literature has illustrated that existing records of an individual’s behaviour upon SNS sites (e.g., likes^3^ and language use^39^) can be informative of personality. Future research may wish to explore alternative labelling strategies and the possibility of leveraging existing recordings of user interaction to compliment the methodologies outlined within this paper; which may lead to the increased performance required for practical applications.

## Data Availability

The code, features and outcome variables for reproducing the results of the machine learning studies is available as a GitHub repository https://github.com/Callum-Woods/Twenty_Seconds_to_Know_You.
